# Gentiopicroside Ameliorates Oxidative Stress and Lipid Accumulation through Nuclear Factor Erythroid 2-Related Factor 2 Activation

**DOI:** 10.1155/2020/2940746

**Published:** 2020-06-16

**Authors:** Meiyu Jin, Haihua Feng, Yue Wang, Siru Yan, Bingyu Shen, Zheng Li, Haiyan Qin, Qi Wang, Jinxia Li, Guowen Liu

**Affiliations:** ^1^Key Laboratory of Zoonosis, Ministry of Education, College of Veterinary Medicine, Jilin University, Changchun, Jilin 130062, China; ^2^Department of Paediatric Hematology, The First Hospital of Jilin University, Changchun, Jilin 130021, China

## Abstract

The activation of nuclear factor erythroid 2-related factor 2 (Nrf2) is closely related to the alleviation of nonalcoholic fatty liver disease (NAFLD) by regulating oxidative stress and lipid homeostasis. Gentiopicroside (GPS), an iridoid glycoside found in the Gentianaceae, possesses anti-inflammatory and antioxidant effects. However, the protective effects of GPS on lipid accumulation and oxidative damage have not been investigated thoroughly in free fatty acid- (FFA-) induced HepG2 cells and tyloxapol- (Ty-) induced hyperlipidemia mice. Cell counting kit-8 assays, Oil Red O staining, Western blotting analysis, extraction of nuclear and cytosolic proteins, and biochemical index assay were employed to explore the mechanisms by which GPS exerts a protective effect on FFA-induced HepG2 cells and Ty-induced hyperlipidemia mouse model. This paper demonstrates that GPS could effectively alleviate NAFLD by elevating cell viability, reducing fatty deposition, downregulating TG, and activating nucleus Nrf2 in FFA-induced HepG2 cells. Meanwhile, GPS significantly regulated the activation of phosphatidylinositol 3-kinase (PI3K)/AKT signaling pathway, Nrf2 antioxidant pathway, peroxisome proliferator-activated receptor *α* (PPAR*α*), and GPS-inhibited sterol regulatory element-binding protein-1c (SREBP-1c) expression in FFA-stimulated lipid accumulation of HepG2 cells and Ty-treated mice. Interestingly, we highlight that PI3K/AKT inhibitor (LY294002) markedly increased the expression of Nrf2 antioxidant pathway, PPAR*α*, and downregulated SREBP-1c in FFA-stimulated HepG2 cells. For these reasons, we found that the deletion of Nrf2 could lose the protective effects of GPS on the Nrf2 antioxidant pathway and PPAR*α* activation and SREBP-1c inactivation in FFA-stimulated HepG2 cells and Ty-treated mice. GPS treatment had no effect on abnormal lipogenesis and antioxidant enzymes in Ty-induced Nrf2^−/−^ mice. This work gives a new explanation that GPS may be a useful therapeutic strategy for NAFLD through upregulation of the Nrf2 antioxidant pathway, which can alleviate oxidative damage and lipid accumulation.

## 1. Introduction

Nonalcoholic fatty liver disease (NAFLD) is a multisystem disease that is the commonest cause of chronic liver metabolic disease in Western countries. NAFLD not only increases the development of cardiovascular, type 2 diabetes mellitus, chronic kidney disease, and cardiac diseases but also increases the morbidity and mortality in patient with liver-related disease [1]. It is well known that NAFLD is an increasingly prevalent and high-incidence disease. In recent years, the “one-hit,” “two-hit,” and “multiple-hit” hypotheses have been used to explain the pathogenesis of NAFLD. And the “multiple-hit” hypothesis provides more precise explanations of NAFLD pathogenesis [2]. The “multiple-hit” hypothesis comprises (1) the insulin resistance, lipotoxicity, and disorder of fat metabolism caused by mitochondrial dysfunction, endoplasmic reticulum stress, and inflammasome activation; (2) the dysfunction of adipose tissue; (3) the genetic determinants; (4) the epigenetic factors; and (5) the dietary factors [2]. Importantly, simple accumulation of lipid plays a key role in the development of NAFLD [3]. Moreover, abnormal lipid metabolism can produce lipotoxicity that induces oxidative stress [4]. Therefore, the present study will focus on the inhibition of the accumulation of lipid and oxidative damage.

In the liver of patients with NAFLD, patients can accumulate fat that is mainly in the form of triglycerides [5]. Triglycerides are synthesized through esterification of free fatty acids (FFAs) and glycerol. There have been studies highlighting FFA that can promote accumulation of lipid-derived toxic metabolites in HepG2 cells [6, 7]. And FFA treatment induced the overexpression of SREBP-1c that might be the main cause of *β*-cell production of lipotoxicity [8]. The previous work on peroxisome proliferator-activated receptor *α* (PPAR*α*) has indicated that PPAR*α* can regulate lipid and glucose metabolism in the treatment of dyslipidemia and diabetes [9]. Importantly, PPAR*α* is crucial for fatty acid metabolic homeostasis in the liver and inhibiting the development of NAFLD [10]. Thus, activating PPAR*α* and decreasing SREBP-1c may contribute to alleviating the lipid accumulation and lipotoxicity and inhibiting the development of NAFLD.

The nuclear factor erythroid 2-related factor 2 (Nrf2) is known as the “main regulator” of the antioxidant response that regulates the expression of hundreds of genes, such as SOD, GSH peroxidases, and catalase [11]. The antioxidant characteristics of Nrf2 can alleviate the development of numerous liver diseases [12–14]. For example, the Nrf2^−/−^ mice promoted the happening of more oxidative stresses that induced the evolution of NAFLD to NASH comparing with the WT mice [15]. Recent studies have shown that many small molecule compounds play the functions of anti-inflammatory, antioxidative stress, and antiapoptosis by activating PI3K/Akt/Nrf2 signal [16–18]. Some studies have found that S-propargyl-cysteine can protect MCD-induced fatty liver by the activation of Akt/Nrf2/HO-1 pathway [19]. Therefore, this study explored whether gentiopicroside (GPS) can improve the lipid toxicity and oxidative stress caused by triglyceride accumulation in hepatocytes through PI3K/Akt/Nrf2 signal.

GPS is extracted from roots and rhizomes of Gentianaceae, and iridoid glycosides are the main active component of GPS. Several researchers have theoretically investigated that GPS has the function of antioxidation and liver protection [20, 21]. However, the association between GPS and PI3K/Akt/Nrf2 signal has not been investigated in NAFLD. In addition, tyloxapol (Ty), a surfactant, can increase triglyceride content in the blood and cause hyperlipidemia [22]. It has been found that Ty can cause the accumulation of triglycerides in the liver [23]. Many studies have used the NAFLD model induced by Ty for drug screening [24]. The current study confirmed that GPS treatment activated the PI3K/AKT and Nrf2 pathway in FFA-stimulated HepG2 cells and Ty-treated mice; however, Nrf2 pathway activation made a contribution to the antioxidant and alleviation lipid accumulation properties of GPS treatment, not the PI3K/AKT/Nrf2 pathway activation. These studies demonstrate that GPS ameliorated lipid accumulation through Nrf2 activation.

## 2. Materials and Methods

### 2.1. Materials

Gentiopicroside (GPS), purity ≥ 98%, was supplied by the Meilun Biotechnology (Dalian, China). Oleic acid, palmitic acid, and tyloxapol (Ty) were bought from Sigma (St. Louis, MO, USA). LY294002, a specific inhibitor of PI3K and AKT, was supplied by Cell Signal Technology (Beverly, MA, USA). TC, TG, ALT, AST, AOC, SOD, and MDA were offered by Jiancheng Bioengineering Institute of Nanjing (Nanjing, China). GSH was obtained from Solarbio Science & Technology (Beijing, China). AKT and p-Aktwere got from Affinity (Cincinnati, OH, USA). Antibodies against PI3K, p-PI3K, HO-1, and NQO1 were acquired from Cell Signal Technology (Beverly, MA, USA). Primary antibodies against Nrf2, *β*-actin, and LaminB came from Proteintech (Boston, MA, USA). GCLC, GCLM, and PPAR*α* were from Abcam (Cambridge, MA, USA). SREBP-1c was obtained from Novus Biologicals (Littleton, CO, USA).

### 2.2. Cell Culture and Treatment

Cell lines of wild-type (WT) HepG2 and Nrf2^−/−^ HepG2 were supported by China Cell Line Bank (Beijing, China) and Dr. Ci Xinxin (Institute of Translational Medicine, The First Hospital, Jilin University), respectively. These cells were grown in 4.5 g/L glucose DMEM (Gibco, NY, USA), 10% fetal bovine serum (FBS, Clark, Australia), and 1% penicillin/streptomycin (Meilun Biotechnology, Dalian, China) in a humidified 5% CO_2_/95% air atmosphere at 37°C. These cells were pretreated with 4.5 g/L glucose DMEM (serum free) for 3 h before treatment.

WT HepG2 cells were exposed to 1 mM FFA (oleate : palmitate = 2 : 1) for different time points from 0 h to 24 h. WT HepG2 cells were incubated in 0, 4, 20, 100, 200, and 500 *μ*M GPS for 24 h. The cells were cultured with GPS for 1 h; after that, the cells were coincubated with 1 mM mixture FFA for 24 h. Cytotoxicity of FFA and GPS was assayed using CCK-8 kits (Invigentech, CA, USA).

### 2.3. Oil Red O Staining

Cells and tissue sections were cleaned with PBS and then fastened with 4% paraformaldehyde (Servicebio, Wuhan, China) for 20-30 min and carbinol for 1 min, respectively. After washing, cells and tissue sections were cultured with 60% isopropanol for 5 min and then dyed in 0.5% Oil Red O staining liquid (Sigma, MO, USA) for 10-20 min. The staining solution of cells and tissue sections was cleaned, then stained with hematoxylin stain (Solarbio Science & Technology, Beijing, China) for 1-2 min. The lipid droplet was observed by a light microscope.

### 2.4. Western Blot Analysis

Proteins were collected from the cells and hepatic tissue using RIPA (Beyotime, China) with phosphatase inhibitors (Thermo, USA) and PMSF (Beyotime, China). The proteins were separated on a 10% SDS-PAGE electrophoresis and then diverted to a polyvinylidene fluoride membrane. Dissected membranes were then sealed with the nonfat milk solution (5% *w*/*v*) for 1 h. After the membranes were adequately incubated with specific primary antibodies at 4°C overnight, the membranes of proteins were further probed with the anti-rabbit or anti-mouse of HRP-conjugated antibodies (Boster, California, USA) for 1 h at room temperature. Finally, the bands of proteins were detected by super sensitive ECL chemiluminescence (Meilunbio, Dalian, China) and band intensities were analyzed using ImageJ gel analysis software.

### 2.5. Extraction of Nuclear and Cytosolic Proteins

The proteins of nuclear and cytoplasm were derived from the WT HepG2 cells using the Nuclear and Cytoplasmic Protein Extraction Kit (Beyotime, China). The extraction procedure is carried out according to the manufacturer's instruction.

### 2.6. Animals and Treatment

WT female C57BL/6 mice were offered by the Liaoning Changsheng Biotechnology (production license: SCXK2010-0001; Liaoning, China). We used Nrf2^−/−^ female C57BL/6 mice that were provided by The Jackson Laboratory (Bar Harbor, ME, USA). They were arranged in SPF environment at 24 ± 1°C (relative humidity: 40%-80%), with a 12 h light and 12 h dark cycle. All mouse studies conformed to the U.S. National Institutes of Health (NIH), the Guide for the Care and Use of Laboratory Animals, and were authorized by the related ethnical regulations of Jilin University.

To estimate the protection effects of GPS in Ty-induced oxidative damage and lipid accumulation, WT mice were stochastically divided into seven groups: control (normal saline), Ty (500 mg/kg), GPS (20, 40, or 80 mg/kg)+Ty, GPS (80 mg/kg), and fenofibrate (100 mg/kg)+Ty. Nrf2^−/−^ mice were divided randomly into control (normal saline) group, Ty (500 mg/kg) group, GPS (80 mg/kg) group, and GPS (80 mg/kg)+Ty group. All animals were hungered for 12 h and then given 80 mg/kg GPS by intraperitoneal injection. After 1 h, mice were treated by intraperitoneal injection of 500 mg/kg Ty for 12 h; after that, mouse serum and liver tissues were collected for the following researches.

### 2.7. Measurement of TC, TG, ALT, AST, AOC, GSH, SOD, and MDA Levels

To measure the accumulation of lipid in cell lysate and mouse serum, the content of TG was measured with commercial assay kits. TC levels in mouse serum were assayed with TC assay kits. The content of ALT, AST, and MDA in mouse serum was determined using standard commercial assay kits, respectively. To examine the antioxidant capacity in mouse serum, commercial kits were used to measure AOC, GSH, and SOD levels.

### 2.8. Statistical Analysis

All data were presented as mean ± SD. One-way ANOVA and Tukey were used to assess statistical significance for multiple comparisons among the different groups. *p* < 0.05 was defined as significant. The statistical analyses and figures were performed using GraphPad Prism 7.

## 3. Results

### 3.1. FFA-Induced Lipid Accumulation and Cytotoxicity and Evaluation of GPS-Induced Cytotoxicity in HepG2 Cells

**Figure 1:**
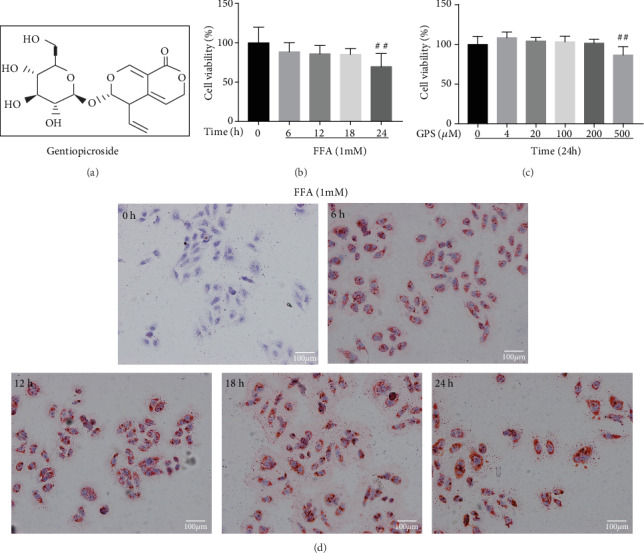
Effects of free fatty acid- (FFA-) stimulated lipid deposition and cytotoxicity and gentiopicroside- (GPS-) induced cytotoxicity in HepG2 cells. (a) The chemical structure of GPS. (b) The cells were subjected to 1 mM FFA mixture (oleate : palmitate = 2 : 1) for five time points (0, 6, 12, 18, or 24 h). Afterwards, cytotoxicity was detected with CCK-8 assay and (d) lipid deposition was observed by Oil Red O staining. (c) The cells were treated with diverse concentrations of GPS (0, 4, 20, 100, 200, and 500 *μ*M) for 24 h. After that, cytotoxicity was evaluated by CCK-8 assay. All data are presented as mean ± SD (three independent experiments). ^##^*p* < 0.01 vs. control group.

In order to evaluate the lipotoxicity model, HepG2 cells were exposed to 1 mM FFA (oleate : palmitate = 2 : 1) for different time points. As shown in Figure 1(b), FFA could induce extremely remarkable cytotoxicity at 24 h. Thus, we investigated FFA-induced lipid storage in cells by Oil Red O staining. Similarly, FFA could induce the most serious lipid accumulation at 24 h (Figure 1(d)). To demonstrate the cytotoxicity of GPS, HepG2 cells were incubated with different concentrations of GPS for 24 h. As described in Figure 1(c), 500 *μ*M GPS could extremely significantly decrease cell viability.

### 3.2. Different Effects of FFA and GPS Activated the PI3K/AKT Signaling Pathway, Nrf2 Antioxidant Pathway, and PPAR*α* in HepG2 Cells

**Figure 2:**
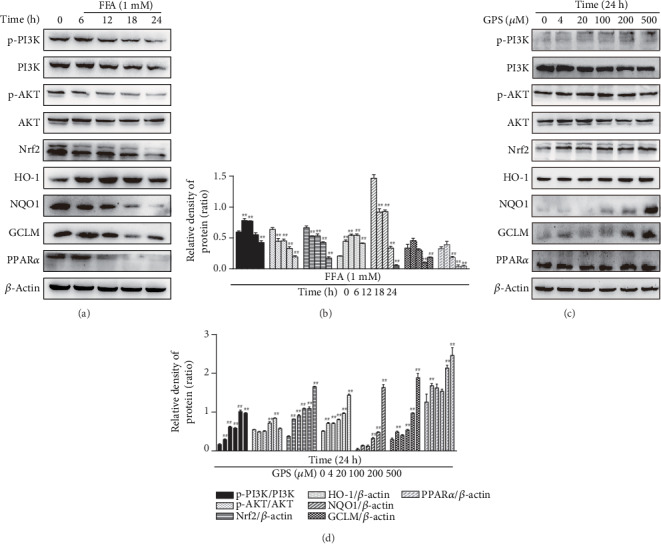
Effects of FFA and GPS exposure on the PI3K/AKT signaling pathway, Nrf2 antioxidant pathway, and PPAR*α* in HepG2 cells. (a, b) All cells were incubated with FFA for 0, 6, 12, 18, or 24 h. (c, d) These cells were subjected to diverse concentrations of GPS (0, 4, 20, 100, 200, and 500 *μ*M) for 24 h. The levels of protein (PI3K/AKT, Nrf2, HO-1, NQO1, GCLM, and PPAR*α*) were assessed by Western blot and quantified by ImageJ software. All data are presented as mean ± SD (three independent experiments). ^#^*p* < 0.05 and ^##^*p* < 0.01 vs. control group.

Previous report that activated the PI3K/AKT/Nrf2 pathway and PPAR*α* is responsible for the improvement of NAFLD, enlightening us to assess the changes of FFA-induced and GPS-induced protein level. Our results unveiled that FFA treatment decreased the PI3K and AKT phosphorylation; inhibited the Nrf2, NQO1, GCLM, and PPAR*α* expression; and elevated the expression of HO-1 in different periods (0, 6, 12, 18, or 24 h). Consistent with previous results, FFA induction caused significant changes of the protein levels at 24 h (Figures 2(a) and 2(b)); however, these effects were strengthened by GPS (0, 4, 20, 100, 200, and 500 *μ*M) in different dosages, especially the dosages of 200 and 500 *μ*M (Figures 2(c) and 2(d)). Our data indicated that GPS may inhibit FFA-induced lipogenesis and oxidative damage in HepG2 cells (Figures 1 and 2).

### 3.3. GPS Treatment Alleviated FFA-Stimulated Lipogenesis, Cytotoxicity, and Oxidative Damage in HepG2 Cells

**Figure 3:**
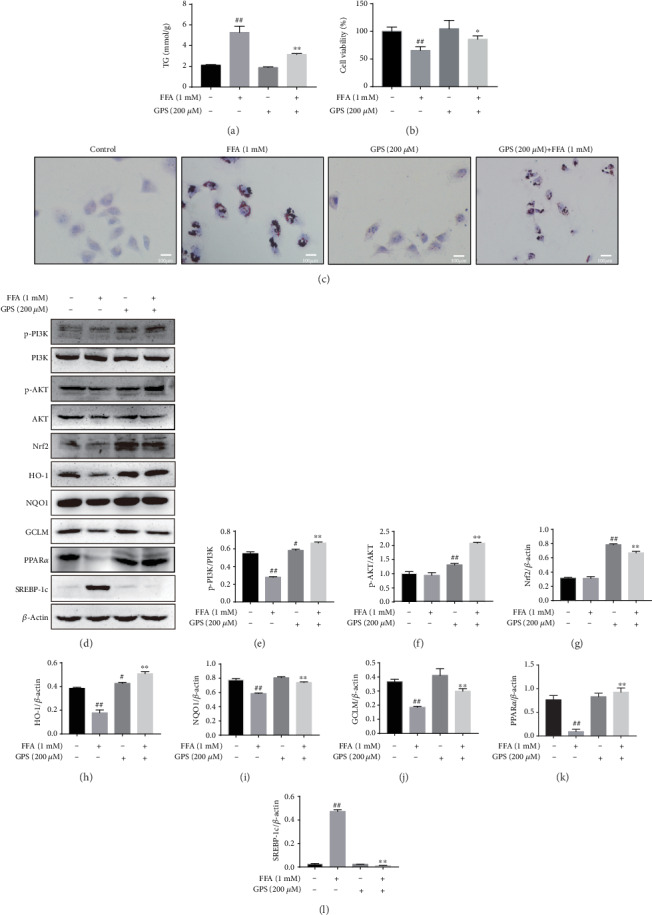
Effects of GPS on FFA-stimulated lipogenesis, cytotoxicity, and oxidative stress in HepG2 cells. These cells were cultured with 200 *μ*M GPS for 1 h and then treated with 1 mM FFA for 24 h. (a) Lipid accumulation was measured using the TG detection kit. (b) CCK-8 assay cell viability. (c) Representative photos of Oil Red O staining. (d) The levels of PI3K/AKT, Nrf2, HO-1, NQO1, GCLM, PPAR*α*, and SREBP-1c were evaluated by Western blotting assays. (e–l) The expression of p-AKT was normalized to AKT; of p-PI3K was standardized to PI3K; and of Nrf2, HO-1, NQO1, GCLM, PPAR*α*, and SREBP-1c was standardized to *β*-actin, respectively. All data are presented as mean ± SD (three independent experiments). ^#^*p* < 0.05 and ^##^*p* < 0.01 vs. control group; ^∗^*p* < 0.05 and ^∗∗^*p* < 0.01 vs. FFA-treated group.

Next, we examined the alleviating effects of GPS on FFA-induced lipid accumulation, cytotoxicity, and oxidative damage in HepG2 cells. Our data demonstrated that 200 and 500 *μ*M GPS treatment could increase phosphorylation of PI3K and AKT as well as elevation of Nrf2, HO-1, NQO1, GCLM, and PPAR*α* in HepG2 cells (Figures 2(c) and 2(d)); however, 500 *μ*M GPS treatment was toxic to HepG2 cells (Figure 1(c)). So, we applied 200 *μ*M GPS to explore its regulated effect on 1 mM FFA-stimulated lipogenesis, cytotoxicity, and oxidative stress in HepG2 cells. Indeed, GPS treatment alleviated FFA-stimulated lipogenesis that is measured by the TG kit and Oil Red O staining (Figures 3(a) and 3(c)). As presented in Figure 3(b), GPS treatment also strengthened FFA-stimulated cell viability. Significantly, we found that GPS pretreatment apparently promoted PI3K and AKT phosphorylation; increased the Nrf2, HO-1, NQO1, GCLM, and PPAR*α* expression; and inhibited the SREBP-1c expression in FFA-stimulated HepG2 cells (Figures 3(d)–3(l)).

### 3.4. Inhibition of Akt Phosphorylation Had No Effect on GPS-Treated Antioxidative Stress and Antilipogenesis in HepG2 Cells

**Figure 4:**
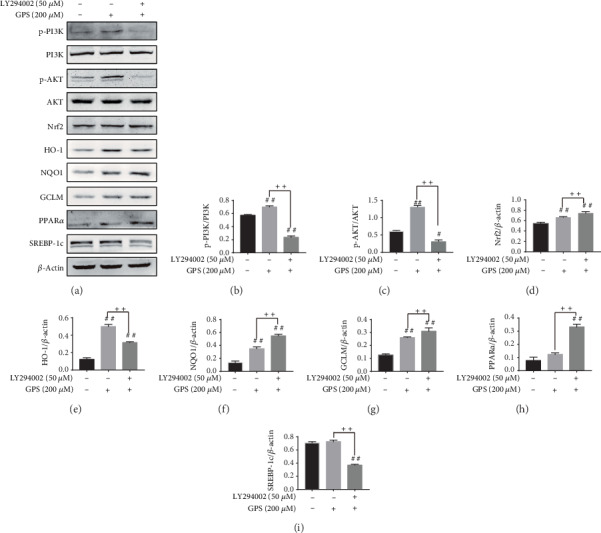
Effects of LY294002 exposure on GPS-treated antioxidative stress and antilipogenesis in HepG2 cells. (a) All cells were subjected to 50 *μ*M LY294002 for 1 h; afterwards, treatment with 200 *μ*M GPS lasts for 24 h. The expressions of PI3K, AKT, Nrf2, HO-1, NQO1, GCLM, PPAR*α*, and SREBP-1c were assayed by Western blotting assays. (b–i) Measurement of p-AKT/AKT, p-PI3K/PI3K, Nrf2/*β*-actin, HO-1/*β*-actin, NQO1/*β*-actin, GCLM/*β*-actin, PPAR*α*/*β*-actin, and SREBP-1c/*β*-actin was quantificated by ImageJ gel software. All data are presented as mean ± SD (three independent experiments). ^#^*p* < 0.05 and ^##^*p* < 0.01 vs. control group; ^++^*p* < 0.01 vs. GPS-treated group.

LY294002 has been generally used as a strong inhibitor by inhibiting the PI3K/AKT pathway [25, 26]. In subsequent studies, we confirmed the interaction between phosphorylation of AKT and Nrf2 in GPS-treated HepG2 cells and examined the cellular protein expression of PI3K, p-PI3K, AKT, p-AKT, Nrf2, HO-1, NQO1, GCLM, PPAR*α*, and SREBP-1c in LY294002-induced and GPS-treated HepG2 cells by Western blot. Surprisingly, Western blot analysis demonstrated that LY294002 inhibited phosphorylation of PI3K and AKT, while Nrf2, HO-1, NQO1, GCLM, and PPAR*α* were significantly upregulated and SREBP-1c was significantly decreased in LY294002-induced and GPS-treated HepG2 cells (Figures 4(a)–4(i)). These findings suggested that PI3K and AKT phosphorylation could not enhance GPS treatment alleviating effects of oxidative stress and lipogenesis.

### 3.5. Deletion of Nrf2 Could Cause GPS-Induced Oxidative Stress and Lipogenesis in FFA-Stimulated HepG2 Cells

**Figure 5:**
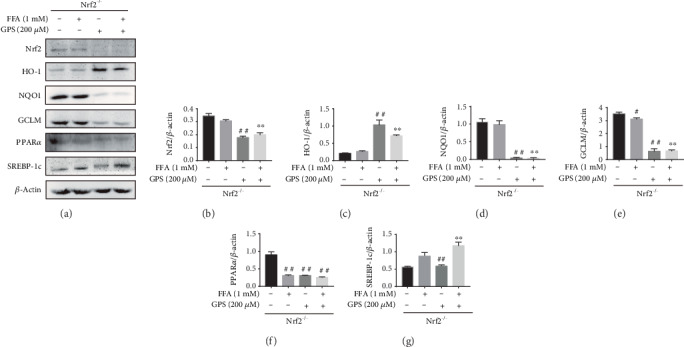
Antioxidative stress and antilipogenesis effects of GPS-mediated Nrf2 on FFA-stimulated lipid deposition in HepG2 cells. Nrf2^−/−^ cells were subjected to 200 *μ*M GPS for 1 h; after that, those cells were incubated with 1 mM FFA for 24 h. (a–g) The expressions of Nrf2, HO-1, NQO1, GCLM, PPAR*α*, and SREBP-1c were detected by Western blot and quantified by densitometric analysis. All data are presented as mean ± SD (three independent experiments). ^#^*p* < 0.05 and ^##^*p* < 0.01 vs. control group; ^∗∗^*p* < 0.01 vs. FFA-stimulated group.

To further elucidate the precise molecular mechanism, we evaluated GPS-induced protective effects of oxidative stress and lipid deposition in FFA-stimulated Nrf2^−/−^ HepG2 cells. Interestingly, the analysis from Western blot indicated that the deletion of Nrf2 inhibited Nrf2, NQO1, GCLM, and PPAR*α* expression in GPS treatment cells (Figures 5(a), 5(b), and 5(d)–5(f)). Deletion of Nrf2 also upregulated SREBP-1c and HO-1 expression in GPS treatment cells (Figures 5(a), 5(c), and 5(g)). These results suggested that deletion of Nrf2 extremely significantly blunted GPS treatment antioxidative stress and antilipogenesis effects, of which the assumption was that Nrf2 activation plays an important role in GPS-treated and FFA-stimulated HepG2 cells.

### 3.6. GPS Treatment Promoted Nrf2 Expression in the Nucleus in FFA-Stimulated HepG2 Cells

**Figure 6:**
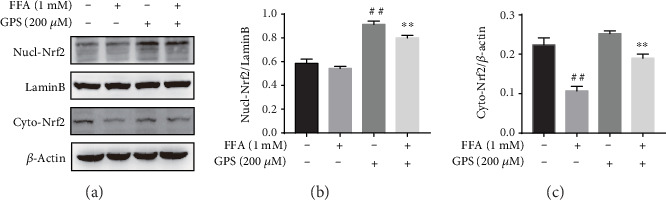
GPS promoted Nrf2 expression in the nucleus. (a) Before 1 mM FFA treatment for 24 h, all HepG2 cells were incubated with 200 *μ*M GPS for 1 h. The nuclear and cytoplasmic protein was extracted by a kit and analyzed by Western blotting. (b, c) Measurement of Nucl-Nrf2/LaminB and Cyto-Nrf2/*β*-actin was quantificated by densitometric analysis. All data are presented as mean ± SD (three independent experiments). ^##^*p* < 0.01 vs. control group; ^∗∗^*p* < 0.01 vs. FFA-treated group.

Because of these findings *in vitro*, our further experiments explored whether GPS could elevate Nrf2 expression in the nucleus in FFA-stimulated HepG2 cells. As expected, GPS treatment markedly increased the FFA-stimulated expression of Nrf2 in the nucleus (Figures 6(a)–6(c)). These results confirmed that GPS may be a Nrf2 activator in FFA-stimulated HepG2 cells.

### 3.7. GPS Treatment Alleviated Oxidative Stress and Hepatic Lipogenesis in Ty-Induced Hyperlipidemia Mice

**Figure 7:**
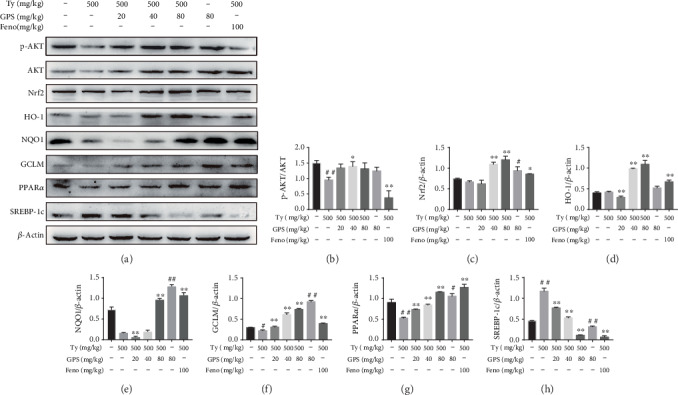
Effects of GPS on the protein levels of AKT, Nrf2, HO-1, NQO1, GCLM, PPAR*α*, and SREBP-1c in tyloxapol- (Ty-) treated mice. The Ty-induced mice were given by intraperitoneal injection with GPS (20, 40, or 80 mg/kg) or fenofibrate (100 mg/kg) for 1 h; after that, injected with Ty (500 mg/kg) for 12 h. (a) Western blot was used to analyze the levels of p-AKT, Nrf2, HO-1, NQO1, GCLM, PPAR*α*, and SREBP-1c in the liver. (b–h) Quantification of p-AKT expression was standardized to AKT and of Nrf2, HO-1, NQO1, GCLM, PPAR*α*, and SREBP-1c was standardized to *β*-actin, respectively. All data are presented as mean ± SD (*n* = 5, number of mice in each group). ^#^*p* < 0.05 and ^##^*p* < 0.01 vs. control group; ^∗^*p* < 0.05 and ^∗∗^*p* < 0.01 vs. Ty-stimulated group.

In order to prove the protective mechanism of GPS treatment in vivo, we investigated whether GPS treatment could inhibit Ty-induced oxidative stress and hepatic lipogenesis in hyperlipidemia mice. Our results demonstrated that GPS treatment markedly elevated AKT phosphorylation and increased protein levels of Nrf2, HO-1, NQO1, and GCLM in Ty-induced oxidative stress mice (Figures 7(a)–7(f)). Consistently, GPS treatment remarkedly activated PPAR*α* and downregulated SREBP-1c in Ty-induced hepatic lipogenesis mice (Figures 7(a), 7(g), and 7(h)). Altogether, these results indicated the protective effects of GPS treatment *in vitro* and *in vivo*.

### 3.8. Deletion of Nrf2 Could Inhibit GPS Treatment Protective Effects in Ty-Induced Hyperlipidemia Mice with Lipid Deposition and Oxidative Stress

**Figure 8:**
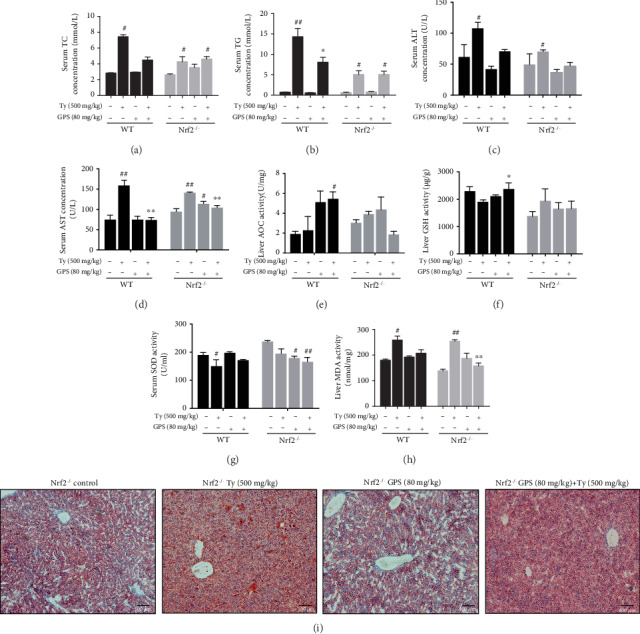
Effects of GPS-mediated Nrf2 on Ty-induced hepatic steatosis and oxidative stress. GPS (80 mg/kg) was intraperitoneal injection to WT and Nrf2^−/−^ mice for 1 h; after that, injected with Ty (500 mg/kg) for 12 h. (a–h) TC, TG, ALT, AST, and SOD in the serum and AOC, GSH, and MDA in the liver were assayed using assay kits. (i) Representative microphotograph of Oil Red O staining of liver tissues (magnification ×100, scale bar = 100 *μ*m). All data are presented as mean ± SD (*n* = 3, number of mice in each group). ^#^*p* < 0.05 and ^##^*p* < 0.01 vs. control group; ^∗^*p* < 0.05 and ^∗∗^*p* < 0.01 vs. Ty-stimulated group group.

To explicit whether the antioxidative stress and antilipogenesis effects of GPS-induced promoted Ty-induced hyperlipidemia mice by Nrf2 activation, we studied the Nrf2 knockout mice. As presented in Figures 8(a) and 8(b), GPS treatment reduced the serum of TC and TG in Ty-induced WT mice; however, these effects were markedly deprived in Ty-induced Nrf2^−/−^ mice. Moreover, to assess the effects of GPS on Ty-induced oxidative damage, the content of ALT, AST, and SOD in the serum and AOC, GSH, MDA in the liver was measured by assay kits. Our results indicated that GPS treatment not only decreased ALT, AST, and MDA levels but also significantly promoted AOC, GSH, and SOD contents in Ty-induced WT mice, whereas the levels of AOC, GSH, and SOD did not increase in Ty-induced Nrf2^−/−^ mice (Figures 8(e)–8(g)). In addition, deletion of Nrf2 had the same effect on ALT, AST, and MDA in GPS treatment and Ty-induced mice (Figures 8(c), 8(d), and 8(h)). In Ty-induced Nrf2^−/−^ mice, result of Oil Red O staining shown that GPS treatment could not inhibit the production of hepatic lipogenesis (Figure 8(i)).

### 3.9. GPS Treatment Protective Effects in Ty-Induced Hyperlipidemia Mice Were Dependent on Activation of Nrf2

**Figure 9:**
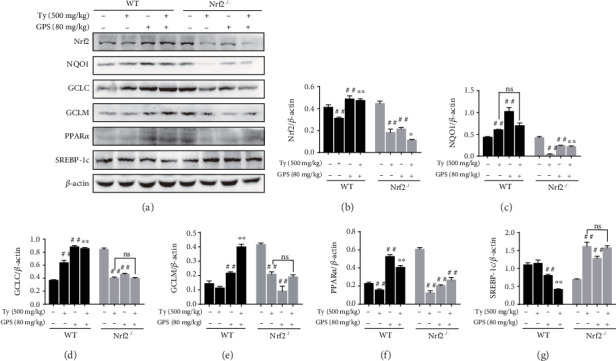
Effects of Nrf2 deficiency on GPS-mediated protein levels of Nrf2, NQO1, GCLC, GCLM, PPAR*α*, and SREBP-1c. (a) WT and Nrf2^−/−^ mice were administered 80 mg/kg GPS for 1 h. After the mice were subjected to 500 mg/kg Ty for 12 h. Western blot examined of Nrf2, NQO1, GCLC, GCLM, PPAR*α*, and SREBP-1c in WT and Nrf2^−/−^ mouse liver. (b–g) Quantification of Nrf2, NQO1, GCLC, GCLM, PPAR*α*, and SREBP-1c was standardized to *β*-actin, respectively. All data are presented as mean ± SD (*n* = 3, number of mice in each group). ^##^*p* < 0.01 vs. control group; ^∗^*p* < 0.05 and ^∗∗^*p* < 0.01 vs. Ty-stimulated group.

To make further efforts ascertain the exact mechanism about the oxidative stress and fatty degeneration of the liver of Nrf2, we investigated the levels of protein expression of GPS treatment in Ty-induced WT mice and Nrf2^−/−^ mice. As presented in Figures 9(a)–9(e), GPS treatment inhibited Nrf2, NQO1, GCLC, and GCLM expression in Ty-induced Nrf2^−/−^ mice but markedly activated in Ty-induced WT mice. Importantly, GPS treatment significantly activated PPAR*α* and downregulated SREBP-1c in Ty-induced WT mice but remarkedly inhibited PPAR*α* and upregulated SREBP-1c in Ty-induced Nrf2^−/−^ mice (Figures 9(a), 9(f), and 9(g)). To conclude, our research results suggested that GPS can effectively alleviate Ty-induced oxidative stress and hepatic steatosis, which may be relied on markedly activation of Nrf2.

## 4. Discussion

The pathogenesis of NAFLD mainly includes intracellular fatty acid accumulation, oxidant stress, ATP depletion, and the function disorder of mitochondria [27]. It is noteworthy that, hepatic lipogenesis is increased, while fatty acid oxidation is insufficient that may promote oxidative stress. And oxidative stress can promote cellular damage and maintain the accumulation of lipids [3]. Nrf2, an oxidative stress-mediated transcription factor, has been suggested to be a target for the treatment of NAFLD [13]. Activation of Nrf2 inhibited FFA-induced oxidative damage [28]. Nrf2 can regulate the oxidant defense systems, mainly including the expression of stress response proteins HO-1; synthesis of reducing factors, such as GSH by GCLC and GCLM; promotion of catabolism of peroxides, and superoxide, such as SOD [29]. There is growing evidence that has demonstrated the key roles of Nrf2 for improving the NAFLD [30, 31]. Nrf2 knockout induced more serious oxidative damage and hepatic lipogenesis in high-fat diet-induced mice [31]. This study set out to determine whether GPS could induce the Nrf2 antioxidant pathway, conferring an antioxidant property to protect FFA-induced HepG2 cells and Ty-induced hyperlipidemia mice. This study found FFA-induced and GPS-induced could inhibit and enhance Nrf2, NQO1, and GCLM expression in HepG2 cells, respectively (Figure 2). Moreover, we illuminated that the deletion of Nrf2 in GPS treatment could inhibit the NQO1 and GCLM expression in FFA-induced HepG2 cells and Ty-induced mice (Figures 5 and 9). Additionally, the activation of HO-1 is independent of the deletion of Nrf2 in GPS treatment and FFA-induced HepG2 cells (Figure 5). This indicated that GPS treatment directly upregulated HO-1 rather than rely on Nrf2 in FFA-induced HepG2 cells. This explained HO-1 activation response to diverse oxidative stimuli [32]. We showed that GPS treatment could upregulate AOC, GSH, and SOD content in Ty-induced WT mice; on the contrary, GPS treatment had no protective effects on the levels of AOC, GSH, and SOD in Ty-induced Nrf2^−/−^ mice (Figure 8). These observations indicated that GPS treatment can activate the Nrf2 antioxidant pathway in FFA-induced HepG2 cells and Ty-induced mice.

Nrf2 activation can be controlled by the PI3K/AKT pathway [17, 33]. S-propargyl-cysteine is reported to promote PI3K/AKT/Nrf2/HO-1 pathway activation, conferring an antioxidative effect to prevent from methionine and choline-deficient diet-induced NAFLD mice [19]. Indeed, this study found that GPS-induced increased the PI3K and AKT phosphorylation in HepG2 cells (Figure 2). Surprisingly, we found that LY294002 inhibited the PI3K and AKT phosphorylation; however, LY294002 promoted Nrf2, NQO1, and GCLM activation PPAR*α* and SREBP-1c inhibition in GPS-treated HepG2 cells (Figure 4). A growing number of studies have highlighted that the activation of the PI3K/AKT signaling pathway is related not only to antioxidant stress but also to antiapoptosis. The apoptosis of hepatocytes in NAFLD is related to the damage of the PI3K/AKT pathway [34]. Cyanidin-3-o-*β*-glucoside protects primary mouse hepatocytes from apoptosis induced by high glucose through the PI3K/AKT pathway [35]. Our previous research shows that GPS treatment was strongly protected against FFA-induced lipotoxicity HepG2 cells through activating the PI3K/AKT pathway and the Nrf2 antioxidant pathway (Figure 3). Our subsequent study found that the deficiency of Nrf2 had not affected the effects of GPS on ALT, AST, and MDA (Figure 8). These results suggested that GPS might inhibited the hepatocyte apoptosis by activating the PI3K/Akt pathway. Unfortunately, we are unable to determine from these data that explained the enhanced antioxidant stress effects of GPS through inhibition of the PI3K/AKT pathway. This problem remains to be solved. Taken together, these data further support that GPS-inhibited oxidant stress may be associated with the Nrf2 activation and not the activation of the PI3K/AKT/Nrf2 pathway.

PPAR*α*, a class of nuclear transcription factor, is activated by ligands that are fatty acid derivatives formed during fatty acid catabolism, lipogenesis, or lipolysis [36]. PPAR*α* can improve steatosis and suppress the development of NAFLD [36]. SREBPs, the lipid synthetic transcription factors, have unique features for fatty acid and cholesterol synthesis [37]. According to the reports that activation of SREBP-1c promoted hepatic lipogenesis and aggravated NAFLD symptoms [38], recently, several reports revealed that regulating PPAR*α* and SREBP-1c could ameliorate NAFLD. Park et al. found that Lonicera caerulea extract could inhibit the expression of SREBP-1c and upregulate PPAR*α* expression in FFA-induced HepG2 cells and in diet-induced obese mice [39]. Importantly, GPS has been shown to upregulate PPAR*α* and downregulate SREBP1 and TG in ethanol-treated HepG2 cells and alcoholic hepatosteatosis mice [40]. Our results similarly discovered that GPS treatment could remarkedly activate PPAR*α* and inhibit SREBP-1c and TG in FFA-induced HepG2 cells and Ty-induced mice (Figures 3 and 7). On the contrary, knockout of Nrf2 produced the reverse results (Figures 5 and 9). This is consistent with that Nrf2 which inhibits lipogenesis by regulating metabolic reprogramming during stress [41]. These results suggest that GPS-mediated Nrf2 can regulate lipid metabolism in FFA-induced HepG2 cells and Ty-induced mice.

In summary, we clarified that the PI3K/AKT pathway could not regulate the Nrf2 antioxidant pathway in GPS treatment. Moreover, our results suggest that HepG2 cells which knockout Nrf2 were susceptible oxidative stress and lipid accumulation in GPS treatment and FFA-induced HepG2 cells, the same as in GPS treatment and Ty-induced Nrf2^−/−^ mice.

## 5. Conclusions

**Figure 10:**
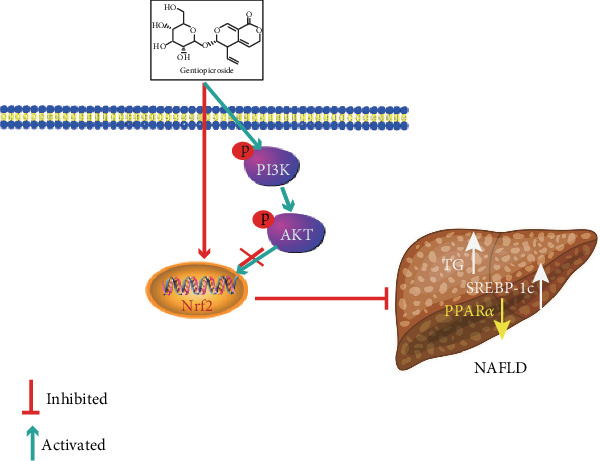
The protection mechanism of GPS-mediated Nrf2 for NAFLD. GPS promoted the phosphorylation of PI3K and AKT and the increase of Nrf2 from cytoplasm to nuclear; however, the PI3K/AKT signaling pathway activation could not help Nrf2 expression in the nucleus and inhibit hepatic lipogenesis.

In conclusion, as shown in Figure 10, the current study demonstrated that GPS treatment strongly protected NAFLD against oxidative damage and lipid accumulation which were dependent on activation of the Nrf2 antioxidant pathway for the first time. This research offers a powerful testimony for the protective effects of GPS in the regulation of oxidative stress and lipid lipogenesis in NAFLD.

## Data Availability

The [DATA TYPE] data used to support the findings of this study are included within the article.

## Abbreviations

AKT: Protein kinase B
ALT: Alanine transaminase
AOC: Antioxidant capacity
AST: Aspartate aminotransferase
CCK-8: Cell counting kit-8
FFA: Free fatty acids
GCLC: Glutamate-cysteine ligase catalytic subunit
GCLM: Glutamate-cysteine ligase regulatory subunit
GPS: Gentiopicroside
GSH: Glutathione
HO-1: Heme oxygenase-1
MDA: Malondialdehyde
NAFLD: Nonalcoholic fatty liver disease
NQO1: NAD (P) H:quinone oxidoreductase
Nrf2: Nuclear factor-erythroid 2-related factor 2
PI3K: Phosphatidylinositol 3-kinase
PPAR*α*: Peroxisome proliferator-activated receptor *α*
SOD: Superoxide dismutase
SREBP-1c: Sterol regulatory element-binding protein-1c
TC: Total cholesterol
TG: Triglyceride
Ty: Tyloxapol.

## Additional Points

*Publisher's Note*. Springer Nature remains neutral with regard to jurisdictional claims in published maps and institutional affiliations.
